# *Ginkgo biloba *for the treatment of vitilgo vulgaris: an open label pilot clinical trial

**DOI:** 10.1186/1472-6882-11-21

**Published:** 2011-03-15

**Authors:** Orest Szczurko, Neil Shear, Anna Taddio, Heather Boon

**Affiliations:** 1 Leslie Dan Faculty of Pharmacy, University of Toronto, Toronto, 130 Dundas St East, Suite 305, Mississauga, ON L5A 3V8, Canada; 2 Department of Medicine, Faculty of Medicine, University of Toronto, Toronto, Canada; 3 Leslie Dan Faculty of Pharmacy, University of Toronto, and Department of Pharmacy and Child Health Evaluative Sciences, The Hospital for Sick Children, Toronto, Canada; 4 Leslie Dan Faculty of Pharmacy, University of Toronto, Toronto, Canada

## Abstract

**Background:**

Vitiligo is a common hypopigmentation disorder with significant psychological impact if occurring before adulthood. A pilot clinical trial to determine the feasibility of an RCT was conducted and is reported here.

**Methods:**

12 participants 12 to 35 years old were recruited to a prospective open-label pilot trial and treated with 60 mg of standardized *G. biloba *two times per day for 12 weeks. The criteria for feasibility included successful recruitment, 75% or greater retention, effectiveness and lack of serious adverse reactions. Effectiveness was assessed using the Vitiligo Area Scoring Index (VASI) and the Vitiligo European Task Force (VETF), which are validated outcome measures evaluating the area and intensity of depigmentation of vitiligo lesions. Other outcomes included photographs and adverse reactions. Safety was assessed by serum coagulation factors (platelets, PTT, INR) at baseline and week 12.

**Results:**

After 2 months of recruitment, the eligible upper age limit was raised from 18 to 35 years of age in order to facilitate recruitment of the required sample size. Eleven participants completed the trial with 85% or greater adherence to the protocol. The total VASI score improved by 0.5 (P = 0.021) from 5.0 to 4.5, range of scale 0 (no depigmentation) to 100 (completely depigmented). The progression of vitiligo stopped in all participants; the total VASI indicated an average repigmentation of vitiligo lesions of 15%. VETF total vitiligo lesion area decreased 0.4% (P = 0.102) from 5.9 to 5.6 from baseline to week 12. VETF staging score improved by 0.7 (P = 0.101) from 6.6 to 5.8, and the VETF spreading score improved by 3.9 (P < 0.001)) from 2.7 to -1.2. There were no statistically significant changes in platelet count, PTT, or INR.

**Conclusions:**

The criteria for feasibility were met after increasing the maximum age limit of the successful recruitment criterion; participant retention, safety and effectiveness criteria were also met. Ingestion of 60 mg of *Ginkgo biloba *BID was associated with a significant improvement in total VASI vitiligo measures and VETF spread, and a trend towards improvement on VETF measures of vitiligo lesion area and staging. Larger, randomized double-blind clinical studies are warranted and appear feasible.

**Trial Registration:**

Clinical trials.gov registration number NCT00907062

## Background

Vitiligo is a depigmentation disorder affecting 1-4% of the world population [1-3]. Fifty percent of cases appear before the age of 20 [4-7], with the disfigurement resulting in psychiatric morbidity in 16 to 35% of those affected [8]. Depression, sleep disturbances, suicidal thoughts, suicidal attempts, difficulties in relationships and avoidance of social situations have been reported in individuals afflicted by vitiligo before adulthood [8,9]. Vitiligo can be confused with leprosy, leading to further stigmatization [10]. The Greater Toronto Area (GTA) has significant Caribbean, African, and South Asian populations, which are doubly at risk. The darker skin makes the vitiligo more apparent, and the presence of lesions similar to endemic communicable diseases carry difficult cultural stigma.

The disease pathogenesis of vitiligo has not been fully elucidated. Autoimmune, biochemical and oxidative stress, genetic, neuronal and environmental factors are thought to interact and contribute to the development of vitiligo [11]. Forschner points to four distinct theories [12]. The first is an "autoimmune hypothesis" supported by the observation that several autoimmune diseases often appear along with vitiligo [12]. In addition, vitiligo sufferers often display elevated levels of serum antibodies to melanocytic antigens (tyrosinase and tyrosinase-related proteins 1 and 2)[11,12]. Second is the "neuronal hypothesis" which states that altered reactions of melanocytes to neuropeptides and catecholamines are responsible for melanocyte destruction [12]. Several studies have found that dopamine can induce apoptosis in human melanocytes [13,14]. The neuronal hypothesis is further supported by the findings that there is close contact between melanocytes and nerve endings in depigmented skin, an observation rarely seen in normal skin [12]. Degenerated and regenerated autonomous nerve fibers and thickened basement membranes of Schwann cells can also be found within vitiligo lesions [12]. Third is the "self-destruct hypothesis", where melanocytes self-destruct due to defects in protective mechanisms responsible for removing toxic melanin precursors [12]. This is thought to lead to the accumulation of melanotoxic indole derivatives and free radicals [12]. Fourth is the "biochemical hypothesis" which postulates an overproduction of a tyrosine hydroxylase cofactor, hydrobiopterin, resulting in increased catecholamine synthesis [12]. This is thought to result in increased reactive oxygen species that are toxic to melanocytes [12]. This is supported by findings of reduced catalase and higher concentrations of hydrogen peroxide in affected and unaffected skin of vitiligo sufferers [12,15].

Conventional treatments for vitiligo include photochemotherapy (PUVA), phototherapy (UVB), vitamin D3 analogues, topical corticosteroids, topical immunomodulators, excimer laser, and surgery. These treatment options have limited success [1,11,12], and some present significant risks, including suspected increases in skin cancer risk by PUVA, skin atrophy with corticosteroids, and skin boils with UVB therapy [1,11,12]. The low benefit to risk ratios of these therapies and the high psychological impact of vitiligo make the search for a safe and effective alternative approach critical.

A systematic review of natural health product (NHP) treatments for vitiligo [16] identified several approaches showing positive results, including topical tocopherol [17], topical vitamin D3[18,19], and oral l-phenylalanine [20,21], and *Ginkgo biloba *[3]. However, the trials were generally of poor quality and the products were often tested as adjuncts to UVA or UVB. One trial using only *Ginkgo biloba *monotherapy in adults showed promising results but was of poor methodological quality.

The specific mechanism of the action of *Ginkgo biloba *in vitiligo is unknown. Parsad points out that ginkgo is known to have anti-inflammatory, immunomodulatory, and antioxidant properties [3], thus potentially impacting the oxidative stress mechanisms of vitiligo. Ginkgo and its constituents have been shown to attenuate oxidative stress in macrophages and endothelial cells [22], scavenge superoxides [23], and protect against UVB-induced toxicity [24].

As stress or anxiety have been postulated to be involved to the mechanisms of vitiligo pathogenesis, ginkgo's anxiolytic effect may also contribute to its use in the treatment of vitiligo. Several studies using animal models report that ginkgo can significantly decrease the detrimental effects of learned helplessness [25], increase intake of novel food in emotional hypophagia, and increase time spent in the open on the elevated plus maze. These effects are similar to those demonstrated for anxiolytic drugs such as diazepam and buspirone [25]. In auditory perturbation stress the detrimental effects of stress indicated by increased errors and increases in plasma concentrations of epinephrine, norepinephrine, and corticosterone were decreased by *Ginkgo biloba *extract EGb 761 [25]. Marcilhac also showed that long term administration of ginkgo extract EGb 761 resulted in decreased basal corticosterone secretion, corticotrophin releasing hormone (CRH) and arginine vasopressin (AVP) gene expression [26]. Under intense surgical stress, CRH, ACTH, and corticosterone plasma concentrations were elevated in control animals, but significantly less so in ginkgo treated animals [26]. Woelk postulates that these findings suggest that EGb 761 modulates the activity of the hypothalamic-pituitary-adrenocortical (HPA) axis [25].

The anxiolytic effect of *Ginkgo biloba *has also been demonstrated in humans. *Ginkgo biloba *has been reported to improve symptoms associated with dementia including anxiety [27]. Jezova showed that ginkgo extract EGb 761 significantly attenuated a stress-induced rise in systolic and diastolic blood pressure, and maintained normal salivary cortisol, while the salivary cortisol increased in the placebo group [28]. Hemmeter et al demonstrated improved sleep efficiency and reduced awakenings with ginkgo intake. In a 107 patient randomized, double blind, placebo controlled 4 week trial Woelk et al demonstrated a dose dependent decrease on the Hamilton rating scale for anxiety (HAMA), indicating an improvement in anxiety [25]. While the mechanism of action of *Gingko biloba *in the treatment of vitiligo is unknown, ginkgo's immunomodulatory, antioxidant, and anxiolytic properties may be of benefit to vitiligo sufferers. The ease of taking an oral pill, the relatively low cost, and the low frequency of adverse reactions with *G. biloba *[29] make its use for vitiligo tempting, but the poor quality of the study and lack of replication of the findings make clinical conclusions difficult. Here we report on a pilot study using *G. biloba *for the treatment of vitiligo in Canadian adolescents and young adults in the GTA using a validated outcome.

## Methods

### Study Design

This prospective, open-label, non-randomized, feasibility trial was conducted in a western suburb of the Greater Toronto Area (GTA) from May to November 2009. The primary objective was to determine feasibility and sample size estimates for a larger randomized controlled trial of ginkgo for vitiligo. The pre-determined criteria used to judge feasibility were:

• successful recruitment of 12 participants in 3 months

• 75% participants completing the trial

• at least 25% of participants achieving a clinically significant 30% repigmentation

• lack of serious adverse reactions requiring hospitalization or threatening the life of participants, as defined by the World Health Organization [30]

### Participants and Setting

We planned to include a convenience sample of male or female participants, aged 12 to 18 years old, with vitiligo vulgaris of any duration as diagnosed by a doctor, and a minimum VASI depigmentation score corresponding to a 3 cm^2 ^completely (100%) depigmented lesion, or a 6 cm^2 ^50% depigmented lesion.

Participants were recruited from the GTA. Study advertisements were made via magazines, posters, on-line, and cultural centers, and via letters to dermatologists and pediatricians in the western GTA. The study was approved by the Health Sciences Research Ethics Board at the University of Toronto (REB), and the Natural Health Products Directorate at Health Canada (NHPD). Informed consent was obtained from all enrolled participants (or their guardians). For all adolescent participants, assent of the participants was sought in addition to parental consent. The conduct of the trial complied with the Helsinki Declaration for studies in humans.

The study took place at a private practice in central Mississauga, a western suburb of the GTA. The study was funded by the Interdisciplinary Network for Complementary & Alternative Medicine Research (IN-CAM). The study product was provided free of charge by Seroyal International Inc. who had no input in the design and conduct of the study nor the interpretation and publishing of the results.

### Study Interventions

The study comprised of 4 visits: initial, and 3 follow up visits every 4 weeks. Total length of treatment was 12 weeks for each participant.

During the initial visit the presence of vitiligo was verified under Wood's lamp. Inclusion and exclusion criteria were assessed, which excluded anyone who had treatment for vitiligo or used of *Ginkgo biloba *within 2 months of study onset or during treatment. Participants' background information, information for the Vitiligo European Task Force (VETF) form [31], Vitiligo Area Scoring Index (VASI)[32], and pictures under incandescent and Wood's lamp were also collected during the initial visit.

A blood sample (6-8 ml) was obtained to determine coagulation status of participants, including complete blood count (CBC), partial thromboplastin time (PTT), and prothrombin time (INR).

At the end of the initial visit, participants were given a 30 day supply of the supplement (Ginkgo plus by Seroyal, Health Canada Natural Product Number 80010368) containing 60 mg of *Ginkgo biloba *(standardized to 15 mg ginkgoflavonglycosides and 4 mg terpene lactones per pill) and instructed to take 1 oral capsule twice per day 10 minutes before breakfast and dinner for the duration of the trial. No other treatments for vitiligo were permitted, but all other medications and medical treatments were allowed and monitored via diaries. Participants were then scheduled for a follow up visit in 4, 8, and 12 weeks.

Sequential follow ups took place every 4 weeks, and included a diary and oral review of other medication use, adverse reactions, and compliance via collection and counting of *Ginkgo biloba *capsules returned. Progression of vitiligo was monitored via the Vitiligo Area Scoring Index (VASI), via the measurement criteria established by the VETF, and via photographs of vitiliginous lesions under incandescent and Wood's light. At the end of each follow up, participants were given a new 30 day supply of *G. biloba*. Orest Szczurko (OS) performed the VASI and VETF assessments after training by Neil Shear (NS). OS was not blinded to treatment allocation. At the end of study at week 12, blood sampling was repeated.

### Withdrawal of subjects

Participants were free to withdraw at any time during the study. Upon indication of withdrawal, participants were asked about the reason for withdrawal, and about any adverse reactions. After withdrawing, participants were not contacted again unless they indicated their wish to receive results of the study by mail.

### Outcome Measures

The **primary outcome measure **was the VASI evaluated at baseline and week 12. The VASI is a validated vitiligo outcome measure [32] that is used to determine the extent of depigmentation. Scores range from 0 (no depigmentation) to 100 (complete depigmentation of the entire body). The VASI takes into account the size and the degree of depigmentation of vitiligo lesions. The VASI score is calculated by multiplying the sum of the area of each lesion by the percent depigmentation of the lesion. The area of each lesion is measured in percent of total body surface using the rule of nines, where the palm plus volar surface of all the digits = 1%. The intensity of depigmentation of each lesion is approximated on a 0-100 percent scale.

**Secondary outcome measures of vitiligo included the VETF score**, digital photographs under incandescent and Wood's light scored at baseline and final week 12 follow up visit. The VETF is designed to evaluate the area of vitiligo lesions, staging, and disease activity. The VETF scoring form includes background information on disease duration, disease activity, family vitiligo history, and degree to which vitiligo impacts the participant's life. It also assesses Fitzpatrick's skin type, presence of thyroid or other autoimmune diseases, and the presence and number of halo nevus. The area of each lesion is measured in percent of total body surface (where the palm plus volar surface of all the digits = 1%). The vitiligo staging score is assessed from 0 to 3 (0 = normal pigmentation, 1 = incomplete depigmentation, 2 = complete depigmentation, 3 = complete depigmentation plus significant hair whitening) and summed for 5 body regions. Thus the staging score range is from 0 to 15. Disease activity is assessed from -1 indicating regressive vitiligo to + 1 indicating progressive vitiligo for each of 5 body regions, thus giving a range from -5 to +5. The VETF and VASI were scored by one of the investigators (OS) after training by a practicing dermatologist, professor and chief of dermatology at the University of Toronto (NS).

All depigmented lesions were photographed with a Panasonic Lumix 9.1 MP digital camera next to a mm/cm ruler.

Adverse events were recorded by participants in a diary, or orally communicated to one of the investigators (OS). At each follow up, the diary was collected and participants were asked: "Since our last visit, did you experience any odd or unusual symptoms?" In the event of an adverse event, patients were instructed to note and record the intensity, timing, and description of any adverse events. The World Health Organizations definition of adverse events was used[30], and correlation with treatment was rated by the clinicians(OS and NS).

### Sample Size Calculation and Statistical Analysis

The primary outcome measure was the difference in VASI scores between baseline and week 12. With an alpha set at 0.05 and beta of 0.20, and assuming a baseline VASI score of 20, a standard deviation of 7 [33], and a 30% improvement, we estimated 10 subjects were required. Allowing for 2 dropouts, 12 participants were deemed sufficient to demonstrate a large treatment effect if present, and demonstrate feasibility of recruitment, retention, safety, and assessment of outcome measures to allow for a calculation of effect size for a larger randomized clinical trial planned for the future. Scores were compared using a paired t-test, where a p-value of < 0.05 was considered significant.

## Results

### Recruitment and follow up

Initial recruitment of participants 12 to 18 years of age proved difficult. After two months of recruitment, the upper age limit was increased to 35 years. Eight participants under 18 and four participants aged 18-35 were recruited (eight female and four male). Eleven participants completed the 12 week study with a mean compliance rate of 87% (range 71%-97%), attended all follow up visits, and were included in the statistical analysis. One female participant dropped out after the first 4 week follow-up, stating a loss of interest in the study.

Participant characteristics are in Table 1.

**Table 1:** Baseline Vitiligo European Task Force characteristics of participants starting treatment

Sex	
Female	8
Male	4
Mean Years of Age (range)	18 (12-29)
Fitzpatrick's phototypes	
I	1
II	0
III	0
IV	2
V	9
VI	0
Duration of disease, mean years (range)	5.96 (2-17)
Ethnicity	
Caucasian	2
South Asian	9
South American	1
Disease activity	
Progressive	7
Non progressive	5
Previous repigmentation	
after treatment	11
spontaneous	0
no repigmentation	1
Depigmentation on scars	5
Stress at onset	2
Stress as precipitating factor	2
Itch before lesions	3
Vitiligo on genitals	3
Thyroid disease	
Yes	1
No	11
Thyroid antibodies	
Yes	0
No	9
unknown	3
Family history of premature gray hair	4
Family history of vitiligo	3
Degree to which vitiligo affects everyday life (out of 10, 10 being the worst) (SD)	3.7 (1.84)
History of autoimmune disease	0
Family history of thyroid or other autoimmune disease	1
Halo Nevus	0

n = 12, SD = standard deviation.

### Efficacy

The status of vitiligo over the study period is displayed in Table 2. The total VASI score showed a significant improvement of 0.5 (P = 0.021) from baseline (4.97) to week 12 (4.47) (Table 2). The trunk, with a VASI score improvement of 0.2 (P = 0.105) and legs, with an improvement of 0.196 (P = .228) showed the greatest responses.

**Table 2:** Vitiligo Area Scoring Index scores at baseline and week 12, n = 11

Area		% area of vitiligo (SD)	extent of depigmentation (0,10,25,50,75,90,100) (SD)	VASI score (SD)
**hands (0-4%)**	**baseline**	1.08 (0.93)	37.27 (35.73)	0.51 (0.56)

	**week 12**	1.08 (0.93)	35.91 (34.27)	0.50 (0.57)

	**change**	0.00 p = 0	-1.36 p = 0.21	-0.01 p = 0.21

**upper extremities (0-18%)**	**baseline**	1.10 (1.69)	34.55 (41.02)	0.70 (1.38)

	**week 12**	1.10 (1.69)	30.91 (35.48)	0.63 (1.18)

	**change**	0.00 p = 0	-3.64 p = 0.20	-0.07 p = 0.35

**trunk (0-36%)**	**baseline**	2.87 (4.04)	69.64 (32.58)	1.66 (1.53)

	**week 12**	2.85 (4.04)	58.64 (32.95)	1.43 (1.57)

	**change**	-0.02 p = 0.36	-11.00 p = 0.11	-0.22 p = 0.10

**lower extremities (0-36%)**	**baseline**	2.12 (2.12)	57.73 (29.78)	1.32 (1.57)

	**week 12**	2.12 (2.13)	50.00 (26.36)	1.13 (1.37)

	**change**	0.00 p = 0.36	-7.73 p = 0.08	-0.20 p = 0.23

**feet (0-6%)**	**baseline**	1.14 (1.41)	50.00 (41.17)	0.79 (1.26)

	**week 12**	1.12 (1.40)	50.00 (41.17)	0.78 (1.26)

	**change**	-0.02 p = 0.36	0.00 p = 0	0.00 p = 0.36

**total VASI (0-100%)**	**baseline**			4.97 (4.74)

	**week 12**			4.47 (4.69)

	**change**			-0.51 p = 0.02

SD, standard deviation.

**Figure 1:**
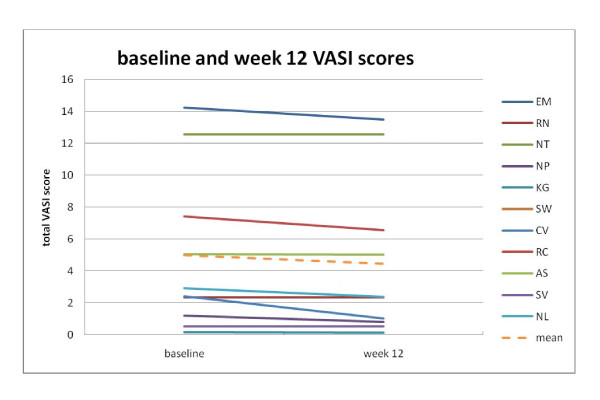
**Baseline and week 12 VASI scores**. Graph of individual participants' Vitiligo Area Scoring Index scores at baseline and end of study (week 12).

The mean percent improvement in the VASI score was 15%, with 2 participants improving greater than 30% and one achieving a 27% improvement in total VASI score. Three others showed improvements in VASI from 11 to 18%. Of the eleven participants who completed the trial, two experienced no change and one experienced a very small 0.4% improvement. Figure 1 charts the baseline and week 12 VASI scores.

The VETF scores for area, staging, and disease activity are shown in Table 3. The VETF scores of total area of vitiligo lesions decreased from 5.91 to 5.56, a decrease of 0.36 (P = 0.102) on the VETF, translating to a 6% decrease in lesion size. The VETF staging score also did not show significant improvement, but did show a trend toward improvement in all body areas, with total staging change improving from 6.5 to 5.8, a change of 0.73 (P = 0.101), or 11%. The treatment had a significant impact on arresting the spread of vitiligo, improving the total spreading score from 2.7 to -1.1, a 3.9 point difference (P =< 0.001).

**Table 3:** The Vitiligo European Task Force scores at baseline and week 12, n = 11

area		% area (SD)	staging (0-3) (SD)	spreading (-1 to 1) (SD)
**head and neck (0-9%)**	**baseline**	0.79 (0.94)	1.55 (0.93)	0.73 (0.47)

	**week 12**	0.73 (0.83)	1.27 (0.90)	-0.36 (0.50)

	**change**	-0.06 p = 0.27	-0.27 p = 0.21	-1.09 p = 0.00

**trunk (0-36%)**	**baseline**	1.33 (1.31)	1.55 (1.04)	0.82 (0.40)

	**week 12**	1.30 (1.30)	1.45 (1.04)	-0.45 (0.52)

	**change**	-0.03 p = 0.21	-0.09 p = 0.36	-1.27 p = 0.00

**arms (0-18%)**	**baseline**	1.81 (2.17)	1.36 (0.81)	0.55 (0.52)

	**week 12**	1.77 (2.06)	1.27 (0.79)	-0.09 (0.54)

	**change**	-0.05 p = 0.36	-0.09 p = 0.36	-0.64 p = 0.00

**legs (0-36%)**	**baseline**	1.99 (2.25)	2.09 (0.54)	0.64 (0.50)

	**week 12**	1.76 (1.76)	1.82 (0.75)	-0.27 (0.79)

	**change**	-0.23 p = 0.26	-0.27 p = 0.09	-0.91 p = 0.02

**totals (0-100%)**	**baseline**	5.91 (4.53)	6.55 (1.92)	2.73 (1.35)

	**week 12**	5.56 (4.27)	5.82 (1.94)	-1.18 (1.25)

	**change**	-0.36 p = 0.10	-0.73 p = 0.10	-3.91 p = 0.00

SD, standard deviation

There were no identifiable differences between the participants who improved the greatest, and those who did not improve at all. Of the three participants that improved the greatest: two had progressive vitiligo, while one had non progressive; two were male, one was female; two had Fitzpatricks skin type 5 and one Fitzpatrick skin type 4. They were 13, 14 and 19 years old, and had suffered with vitiligo for 10, 14 and 2 years and had started study treatment on July 20, July 27, and June 11 2009 respectively. One had vitiligo on hands, the others did not. Two had vitiligo on the torso and all three had vitiligo on the lower body but only one on feet.

In contrast, of the three participants who did not improve significantly: two were female; they were aged 12, 16 and 22 with disease duration of 4, 6 and 9 years and started study treatment on June 9, June 9, and July 16, 2009 respectively. Two had non progressive vitiligo, and one progressive. Two had Fitzpatricks skin type 5 and one type 4. All three had vitiligo on hands; two had vitiligo on upper extremities. All three had vitiligo on the torso area as well as the lower extremities and the feet.

### Safety

Serum coagulation parameters measured at baseline and week 12 (Table 4) demonstrated no significant changes.

**Table 4:** Blood coagulation parameters at baseline and week 12, n = 11

	Platelet count *	range	APTT **	range	INR ***	range
**baseline**	262	184-412	36.5	28-53	1.15	1.0-1.5

**week 12**	284	173-441	34.6	26-40	1.15	1.0-1.3

* Platelet count test range 145 - 400

** APTT test range 26 - 37s

*** INR test range 0.9 - 1.3

One case of watery diarrhea of mild intensity that developed 43 days after starting the *Ginkgo biloba *was reported by a 13 year old male participant. The participant continued to take the *Ginkgo biloba *and the diarrhea resolved within 24 hours without any treatment or protocol deviations. The participant reported having a similar one day bout of watery diarrhea two months before entering the study, when not taking any supplements. The participant, his parents, and the study coordinator agreed that the watery diarrhea episode was likely unrelated to the *Ginkgo biloba *supplement. There were no other adverse events reported by participants.

## Discussion

In this pilot trial, we did not demonstrate feasibility according to the four criteria of recruitment set-out a-priori: 12 participants in 3 months, 75% retention, 25% of participants achieving 30% repigmentation based on the VASI, and lack of serious adverse reactions. The primary challenge for achieving feasibility was the recruitment of 12 participants aged 12 to 18 years old in the specified time period. The initial recruitment was limited to pediatric patients since 50% of vitiligo cases appear before 20 years old, and vitiligo is known to have significant psychological impact. It could be argued that the adolescent years are some of the most psychologically fragile, where looks and social pressures have the most impact, thus the adolescent population was targeted for this study as the need to treat their vitiligo was perceived to be the greatest. Although many people inquired about the study, we had difficulty recruiting the young participants specified in the inclusion criteria. After two months of recruitment, the upper age limit was raised to 35 years old, and this resulted in achievement of the required sample size. Eleven of 12 participants, or 92%, completed the trial.

Although our findings are not conclusive, they do provide preliminary evidence that ginkgo may have a role in the management of vitiligo. Two participants achieved greater than 30% improvement in VASI, with another achieving 27%, and three more 11 to 18% improvement. The percentage of improvement of VETF scores of the three most improved participants were 6,7, and 29% on area; 0,40, and 50% on staging; and 100, 150, and 133% on spread (the spreading measure ranges from -5 when the vitiligo is spreading to +5 when it is repigmenting thus allowing greater than 100% improvement).

Our findings are consistent with a previous report showing effectiveness in treating vitiligo with *Ginkgo biloba*[3]. In a double blind randomized trial conducted in India, Parsad et al reported that 40 mg of *Ginkgo biloba *three times per day for 6 months arrested the spread of vitiligo in 20 out of 25 participants in the active group, and induced marked (75% or greater) repigmentation in 10 of those participants.

Several factors could account for the difference in the magnitude of effect between the present study and the study by Parsad [3]. First, the duration of treatment in our study was 12 weeks, compared with 6 months. Secondly, we were unable to utilize exactly the same *Ginkgo biloba *extract used by Parsad, though both extracts were standardized to the same type and amount of ginkgoflavonglycosides. Third, we utilized 60 mg of *Ginkgo biloba *twice per day, while the Parsad used 40 mg three times per day. While the daily dosage of the standardized ginkgoflavonglycosides and *Ginkgo biloba *is the same, there could be a benefit to taking the *G. biloba *in more frequent doses. It is also possible that genetic differences between the populations of the two studies had an impact, although this is unlikely as 9 of the 12 recruited participants in this study were of South Asian origin. As our study was performed in Toronto and the other in India, dietary and social differences between the two populations might have affected the results. The most likely confounding factor, however, is sun exposure, as it can stimulate the proliferation of melanocytes and affect the production of vitamin D, both of which can impact vitiligo [1,34]. An attempt was made to compensate for this by starting the study in May, but some participants were recruited late in the summer, and completed the 12 week study in November, thus experiencing substantial decreasing intensity of sun exposure over the last few months of their treatment.

Most studies of vitiligo treatment with phototherapy set a 75% repigmentation rate as cosmetically acceptable, and are able to achieve it in 12.5 to 75% of patients after one year of treatment [35]. By comparison, other studies utilizing the VASI as the primary outcome measure have found a 43% improvement with narrow band UVB therapy [32]. Thus a 15% improvement in VASI after 3 months of treatment is much smaller than can be achieved with other therapies over a longer duration.

There were no adverse events attributed to the study herb, and *Ginkgo biloba *did not adversely affect serum platelet, PTT, or INR coagulation parameters. Given the inclusion of adolescent participants in this study, much care was taken to monitor adverse events experienced due to the taking of *Ginkgo biloba*. The most concerning was the potential of *Ginkgo biloba *to reduce coagulation [36-40]. To ensure participant safety and to inform the impact of *Ginkgo biloba *on coagulation parameters, the baseline and week 12 serum platelet count, PTT, and INR levels were measured. There was an unexpected high proportion of participants with elevated PTT levels at baseline (3 of 11). The PTT showed a decreasing trend toward normalization by the end of the study. It is also interesting that the mean platelet count increased by week 12, moving opposite of the expected anticoagulant effect of ginkgo, while the INR remained the same. Our results suggest that *Ginkgo biloba *did not have a negative impact on these blood clotting parameters. The impact of *Ginkgo biloba *on serum coagulation was not the primary outcome of our study, thus the study was not powered to evaluate the significance of these results.

As one of the top selling herbal medicines in the United States [41], and accounting for 1% of total prescriptions in Germany [39], the use of *Ginkgo biloba *for vitiligo is appealing due to the ease of use of taking an oral readily available over the counter product compared with frequent and lengthy ultraviolet phototherapy sessions, and the relatively inexpensive cost of the herbal supplement when compared to phototherapy [42]. Further, the relatively good safety profile of gingko [29] make it appealing for self administration by vitiligo sufferers. However, it is recommended that any attempt to use ginkgo in the management of vitiligo should be carefully monitored by a health care practitioner given that there are still many questions about the correct dose, its true effectiveness, interactions with other conditions or therapies, and possible adverse reactions.

## Limitations

There are several limitations to our pilot study that warrant discussion. The purpose of this study was to assess the feasibility of studying ginkgo for the management of the vitiligo in a larger, blinded randomized controlled trial and as such this study was not designed to allow us to draw any conclusions about its efficacy. The sample size was small, thus our findings are preliminary and definite conclusions about the efficacy and safety of ginkgo cannot be made. Because the study was open labeled, not controlled, and not randomized, it is quite likely that some of the demonstrated benefit may be due to a placebo effect. However, this is unlikely that all of the results we report can be accounted for by spontaneous repigmentation of vitiligo because these are generally considered clinically insignificant and cosmetically undetectable [34,43].

Our primary outcome measure, the VASI, has a degree of subjectivity associated with it. We attempted to strengthen the objectivity of the outcomes by using a trained assessor, and by verifying assessments using digital photographs. However, both the VASI and VETFI require the assessor to approximate both the area of vitiligo lesions and their intensity. Since the assessor was not blinded to treatment in this open-label pilot study, it is possible that bias was introduced in the outcome assessment.

The young age of the participants may also have impacted the results, as younger vitiligo sufferers can respond more quickly to treatment. It is possible that the relatively smaller lesion sizes and durations in our participants could have resulted in over-estimating the effectiveness of treatment [44]. The *Ginkgo biloba *extract itself could have been a source of variability inherent in studying herbs, but care was taken to ensure that the herb brand chosen was standardized to ginkgoflavonglycosides and terpenoids, both being good measures of *Ginkgo biloba *quality. Despite these limitations, we have estimates of effectiveness that can be used to estimate a sample size for a larger study. And the results are compelling enough to proceed with a larger trial.

## Conclusions

This pilot study focused on determining the feasibility of a future large randomized controlled trial of ginkgo for the treatment of vitiligo. Due to the difficulties of recruitment of 12 to 18 year old participants, the study as originally conceived would not have been feasible. However, with the expansion of participants from 12 to 35 years old, our findings indicate that a larger study is feasible and ginkgo is worth investigating further as a potential treatment of vitiligo. Given the limited usefulness of current treatments and the frequency of treatment visits required for phototherapy, an over the counter herb supplement is a welcome possibility for vitiligo sufferers. Given that our small study found statistically significant improvement on some secondary outcome measures, a larger methodologically rigorous double-blind placebo-controlled randomized clinical trial is recommended.

## Abbreviations

VASI: Vitiligo Area Scoring Index; VETF: Vitiligo European Task Force; GTA: Greater Toronto Area; PUVA: psoralen with ultra violet light A; UVB: ultra violet light; NHP: natural health product; IN-CAM: Canadian Interdisciplinary Network for Complementary & Alternative Medicine Research; CBC: complete blood count; PTT: partial thromboplastin time; INR: prothrombin time.

## Competing interests

The study was funded by the Interdisciplinary Network for Complementary & Alternative Medicine Research (IN-CAM). The study product was provided free of charge by Seroyal International Inc. who had no input in the design and conduct of the study nor the interpretation and publishing of the results.

The authors declare no competing interests.

## Authors' contributions

OS conceived of the study, carried out the data collection and review, and drafted the manuscript. HB conceived of the study, guided the design, helped to resolve methodological concerns, and critically revised the manuscript. AT & NS guided the design, helped to resolve methodological concerns, and critically revised the manuscript. All authors read and approved the final manuscript.

## Pre-publication history

The pre-publication history for this paper can be accessed here:

http://www.biomedcentral.com/1472-6882/11/21/prepub
